# Bioinspired algorithm based on Physarum polycephalum for the formation of decentralized mesh networks in multi-robot systems

**DOI:** 10.1038/s41598-025-33456-y

**Published:** 2025-12-26

**Authors:** Dieisson Martinelli, André Schneider de Oliveira, Vivian Cremer Kalempa

**Affiliations:** 1 Department of Information Systems, Universidade do Estado de Santa Catarina (UDESC), São Bento do Sul, 89283-081 Brazil; 2 Graduate Program in Electrical and Computer Engineering, Universidade Tecnológica Federal do Paraná (UTFPR), Curitiba, 80230-901 Brazil

**Keywords:** Multi-robot systems, Physarum polycephalum, Decentralized system, Engineering, Mathematics and computing

## Abstract

This work proposes a bio-inspired approach for decentralized coordination in multi-robot systems, applied to a simulated port scenario. The methodology integrates the Robot Operating System (ROS) with the Stage simulator, enabling modeling of a port environment with three autonomous robots, each capable of navigation and obstacle avoidance. The main contribution is a connectivity module inspired by Physarum polycephalum, which manages the mesh network and allows decentralized task sharing whenever connections exist. The algorithm adapts continuously to robot movement and environmental changes, ensuring efficient communication when possible and autonomous operation when disconnected. Experiments confirmed that robots relying only on local perception can form and maintain a functional network. Results showed connections established in less than two seconds on average and reconfigured almost instantly after fault, demonstrating resilience. New robots were integrated in only 0.092 seconds on average, validating scalability. A comparison between scenarios with and without communication revealed a 17.87% efficiency improvement, reducing execution time from 621 to 510 seconds thanks to dynamic load balancing. In summary, the study demonstrates the feasibility of a bio-inspired solution for decentralized coordination in multi-robot systems, capable of generating efficient, resilient, and adaptable communication networks, essential for cooperation in real-world environments. A demonstration video is available at https://youtu.be/ZGPswbfeRKA.

## Introduction

Multi-robot systems (MRS) have become a practical option for complex logistics scenarios such as ports, factories, and distribution centers, where fleets of mobile robots must coordinate navigation, task allocation, and resource sharing under uncertainty and frequent topology changes^1,2^. However, the simultaneous operation of multiple robots in dynamic environments imposes challenges of communication and coordination, especially without centralized infrastructure. In these settings, communication is often intermittent due to occlusions, interference, and mobility. In port contexts, the adaptive formation of networks and the decentralized sharing of information are critical to ensure cooperation and overall system performance.

Traditional network protocols, such MANET protocols (AODV and OLSR) provide proven routing mechanisms, yet their control overhead and route maintenance degrade under high mobility and rapidly changing link quality, limiting their effectiveness for tightly coupled robot coordination^3^. Bio-inspired strategies (ACO and PSO) have improved coverage and energy efficiency in MRS, but typically rely on sensitive parameter tuning or continuous traffic to sustain performance^4^.

In this scenario, Physarum polycephalum emerges as a promising alternative. This unicellular organism forms robust and adaptive transport networks from local stimuli, reinforcing useful connections and retracting little-used routes^5^. Such properties make it suitable for multi-robot systems, where topology changes rapidly and there is no time or resources to maintain global routing information. Recent computational models show that simple sensing–movement–deposition rules can generate stable and resilient emergent networks^6^.

Despite these advances, few studies have directly applied the emergent behavior of Physarum polycephalum to the communication of mobile robots, considering real metrics such as signal strength (RSSI) or link stability. This work leverages these principles for communication maintenance by using Physarum-inspired local rules to infer, strengthen, or prune robot-to-robot links based on signal quality and stability, thus enabling decentralized task sharing only when links are truly viable.

In summary, this work moves beyond establishing connectivity to demonstrating how bio-inspired mechanisms can optimize decentralized coordination. The main contributions of this study are threefold:

- **A Physarum-inspired Connectivity Layer:** We propose a decentralized mechanism that uses local trail reinforcement to filter stable communication links. By prioritizing reinforced paths, this layer acts as a topology control mechanism that mitigates the complexity of traditional flooding-based discovery, enabling scalable task sharing.
- **Resilient Mesh Formation:** We demonstrate that the algorithm supports instantaneous reconnection and rapid scalability, confirming its robustness for dynamic environments without central oversight.
- **Efficiency in Task Allocation:** We provide quantitative evidence that coupling this bio-inspired layer with a decentralized task-sharing protocol reduces the total execution time compared to isolated operation, validating the approach for practical logistics scenarios.

Although this work does not directly implement or integrate existing MANET protocols, the proposed Physarum-inspired connectivity mechanism can conceptually operate as a complementary layer to these frameworks. By providing locally inferred link stability and dynamic neighbor weighting, it could inform routing decisions or QoS configurations in traditional robotic communication stacks, suggesting a promising direction for future integration.

## Related work

Recent advances in decentralized coordination of MRS have demonstrated robust solutions in dynamic scenarios, highlighting distributed task allocation algorithms, consensus mechanisms, and fault-tolerant strategies. However, most of these studies assume stable communication as a premise, leaving open the issue of adaptive connectivity maintenance.

Decentralized coordination has proven feasible even under adverse conditions, leveraging distributed consensus, resilience, and scalability. For example^7^, proposed MURD-TAP, based on ADMM consensus, achieving optimal task allocation without a central controller, but requiring topological stability for convergence. In^8^, a dynamic strategy was introduced that considers robot capacity and energy, adapting to load variations, though still presupposing continuous connectivity. Meanwhile^9^, proposed fault-tolerant coordination, enabling isolated robots to operate autonomously, while^10^ introduced CoLoSSI, which maintains incremental allocation even under temporary network fragmentation. Systematic reviews such as^11^ confirm these advances but emphasize that adaptive communication remains underexplored.

In the bio-inspired field, several works explore properties such as self-organization, resilience, and cooperation. Among the most applied models are ACO algorithms^12,13^, PSO^14,15^, Firefly^16,17^, and ABC^18^, achieving improvements in coverage, energy efficiency, and collision avoidance. Other proposals, such as^19^, draw inspiration from bacterial colonies for collective gas mapping, reinforcing the potential of nature as a source of distributed strategies. Within this spectrum, Physarum polycephalum has stood out since the seminal model of^20^, with applications in route planning^21,22^ showing gains in trajectory smoothness and energy efficiency.

Unlike the aforementioned approaches, which often rely on precise parameter tuning, continuous message exchange, or centralized supervision for convergence, these strategies still exhibit limitations when applied to highly dynamic environments. Consensus-based models, although mathematically elegant, depend on persistent connectivity to ensure stability; swarm-based methods such as ACO and PSO can adapt to local changes but tend to generate redundant traffic and are sensitive to communication noise; and hybrid optimization algorithms frequently increase computational cost, reducing real-time applicability. In contrast, Physarum polycephalum naturally balances exploration and exploitation through local reinforcement and retraction mechanisms, maintaining global coherence without requiring centralized coordination or continuous information exchange.

Therefore, the main advantage of adopting a Physarum-inspired model lies in its intrinsic adaptability and simplicity. The organism’s behavior demonstrates that complex network structures can emerge from minimal local rules, allowing the system to reorganize autonomously in response to environmental and topological changes. This feature directly addresses the limitations observed in previous methods, providing a biologically grounded mechanism for maintaining connectivity and coordination in mobile multi-robot systems operating under unstable communication conditions.

Beyond bio-inspired coordination algorithms, several communication paradigms from other domains have been adapted to robotic systems, such as Vehicular Ad Hoc Networks (VANETs), Internet of Things (IoT) architectures, and Wireless Sensor Networks (WSNs)^23–25^. Although these frameworks provide valuable insights into distributed communication, they typically assume stable connectivity, hierarchical organization, or periodic route maintenance. Such assumptions become limiting in highly dynamic multi-robot environments. In contrast, the Physarum-inspired approach proposed in this work establishes and maintains connectivity solely through local interactions and environmental feedback, eliminating the need for global synchronization or centralized control. This emergent mechanism directly addresses the limitations observed in these traditional models, offering a lightweight and self-organizing alternative suitable for mobile robotic networks.

## Physarum-inspired algorithm

**Fig. 1:**
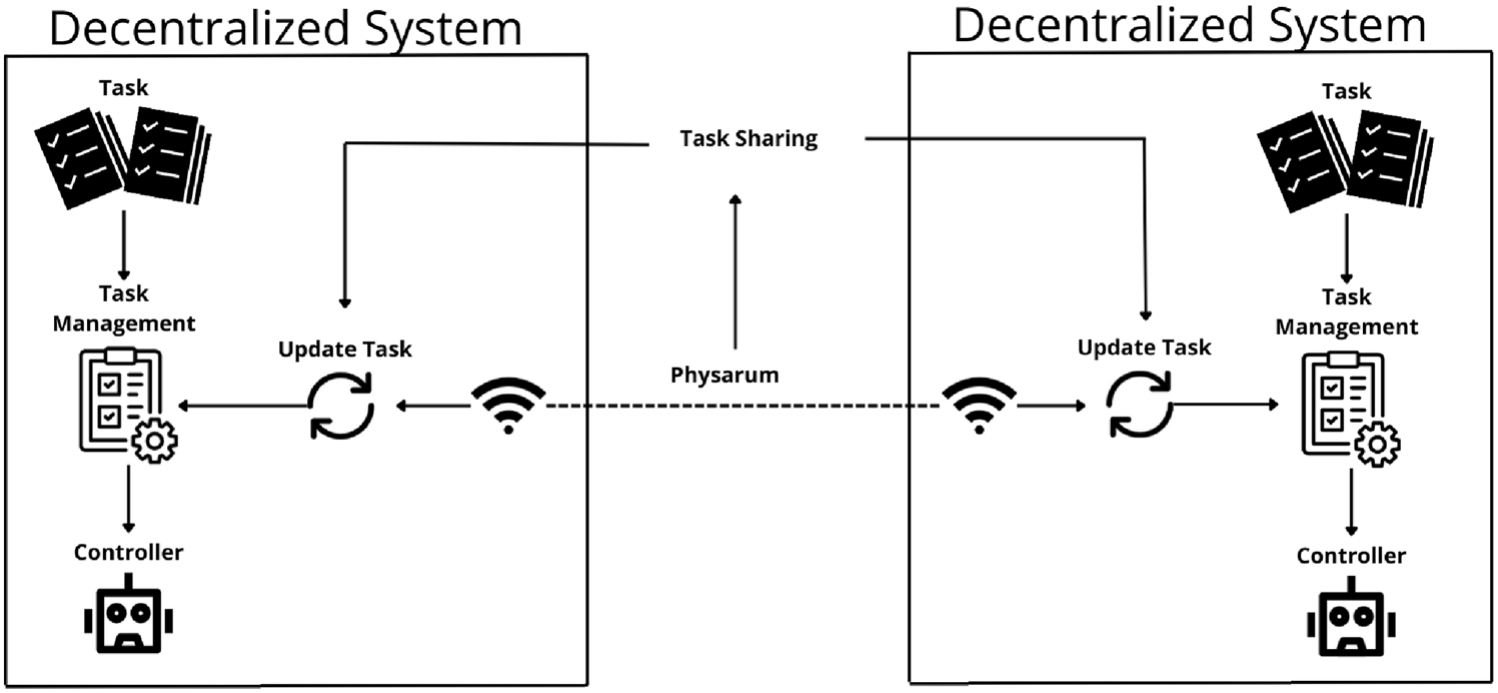
Example of the system architecture.

The proposed system was developed with the objective of simulating a logistics operation environment with multiple autonomous mobile robots, integrating container pickup and delivery tasks. The system architecture was designed to be decentralized, modular, and bio-inspired, allowing each robot to operate autonomously while exchanging information only with nearby robots, through a connectivity network based on the behavior of Physarum polycephalum. The general architecture of the system can be seen in Fig. 1.

In this architecture, each robot operates independently, receiving its tasks through a ROS topic, locally managing its task queue, and executing control commands to move to the container pickup and delivery points. However, through the algorithm inspired by Physarum polycephalum, it is possible to detect network connections based on signal intensity between robots. When such a connection is established between two robots, mutual task sharing and updating of their respective local management lists occur, promoting more robust decentralized coordination.

### Bio-inspired connectivity layer

In its individual operation, each robot applied to this architecture was designed to function completely autonomously, without relying on a central server or external supervisor. Upon receiving a new task, the robot inserts it into its local task queue, which operates as a priority list based solely on arrival order.

Task management is performed locally. The robot continuously evaluates its task list and selects the next action based on its current state, if it is not executing another task, it retrieves the next available one. Once the active task is defined, the motion planning module employs autonomous navigation algorithms to generate the route to the corresponding pickup or delivery point. For this purpose, the ROS *move_base* package is used, integrating global planning algorithms, such as Dijkstra, with local planners and obstacle avoidance mechanisms, enabling the robot to autonomously navigate the environment. During this process, the robot also relies on onboard sensor data, such as Light Detection and Ranging (LiDAR), to update the local map and detect dynamic obstacles. This allows dynamic adjustments to the planned route, avoiding collisions and ensuring safe navigation even in dynamic or partially unknown environments.

**Fig. 2:**
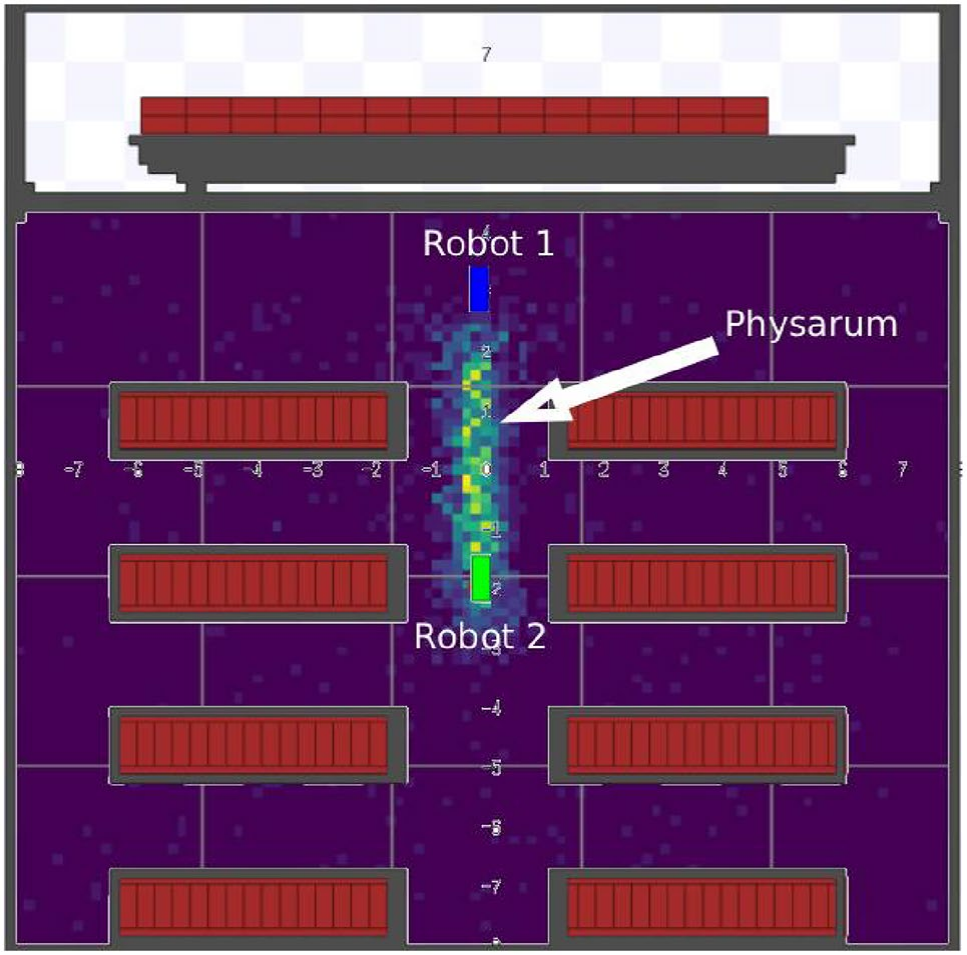
Execution of the Physarum Polycephalum algorithm by robot 2.

However, although the robots are capable of operating independently, the proposed architecture introduces a mechanism of connection and cooperation among the robots that is based on bio-inspired principles, specifically on the behavior of Physarum Polycephalum. In this context, the algorithm inspired by Physarum Polycephalum is used to simulate the formation of network connections between robots based on signal intensity. Each robot periodically emits a signal that is interpreted as a food source for the Physarum Polycephalum. Each robot executes the algorithm based on Physarum Polycephalum, which explores the environment in search of a food source, in this case, the signal of other robots. When the signal is found, the trail is reinforced and a connection is established. A representation of this process can be observed in Fig. 2.

From that moment on, the connected robots initiate an information exchange protocol. One of the main objectives of this exchange is the sharing and synchronization of task lists. Each robot compares its local list with that of the other, removing duplications, redistributing tasks in a balanced way, and updating the status of the shared tasks. This decentralized synchronization allows the system as a whole to behave more efficiently and adaptively, especially in situations where the workload is unbalanced among robots.

In summary, the proposed architecture enables each robot to fully function as an autonomous unit, while simultaneously fostering the emergence of coordinated collective behaviors through a network layer inspired by biological systems.

The proposal of this work stems from the observation of the remarkable adaptive and optimization capabilities demonstrated by Physarum Polycephalum, capable of solving complex problems in a decentralized manner. Classical and recent studies have shown that this organism can find shortest paths in mazes, connect multiple food sources efficiently, and reorganize its tubular networks in the face of environmental changes^5,26^.

### Computational model of the physarum polycephalum

Inspired by the properties of Physarum Polycephalum, a decentralized connectivity algorithm was developed that computationally simulates the behavior of Physarum Polycephalum to establish connection routes between mobile robots in a simulated environment. The algorithm operates through an agent-based approach, in which each agent represents a discrete portion of the organism’s living mass. These agents interact locally with the environment, perceive stimuli (such as chemical trails and food), move adaptively, and reinforce paths, allowing the emergence of a network of connections among nearby robots, even in scenarios with limited or intermittent communication.

The similarity between the computational model and the biological organism can be observed in several fundamental parallels: the cytoplasmic flow of Physarum Polycephalum, characterized by pulsatile movements that distribute the cytoplasm, is represented by discrete mobile agents that move continuously, simulating the cell’s internal transport, chemical sensing, through which the organism detects gradients of nutrients or repellents, is translated into virtual sensors that allow agents to perceive local values of food, trail, and mass, tube reinforcement, associated with the thickening of frequently traversed regions, corresponds to trail deposition that strengthens commonly used paths, the retraction of inactive regions, which in the organism results in metabolically inert areas, is modeled by the death of agents that enter a hibernation state, and finally, adaptive expansion, observed in the organism’s growth front, is simulated by the selective reproduction of agents at the edges of the mass, especially in regions of high activity.

Each agent is defined by a set of attributes that regulate its local dynamics:

- (*x*, *y*): continuous coordinates on the map.
- $\theta$: direction of movement (*heading*), in radians.
- *s*: internal strength or energy, representing its vitality.
- *r*: role in the system, which can be *explorer* or *follower*.
- $t_{\text {hunger}}$: time since the last feeding.

The behavior of the agent is defined by its role: explorers perform more stochastic movements and long-range sensing, promoting the discovery of new paths, while followers reinforce known regions. At each iteration, the position (*x*, *y*) is updated according to Equation 1, where *v* is the constant scalar velocity and $\theta _t$ is the orientation angle at instant *t*, adjusted according to the stimuli perceived. This adjustment is performed by incremental rotations (left, right, or maintaining the current direction), based on assigned scores, so that the trajectory emerges from the continuous interaction with the environment.

1 $$\begin{aligned} \begin{aligned} x_{t+1}&= x_t + v \cdot \cos (\theta _t) \\ y_{t+1}&= y_t + v \cdot \sin (\theta _t) \end{aligned} \end{aligned}$$

Where $x_t$ and $y_t$ represent the current position of the agent along the horizontal and vertical axes, respectively; $x_{t+1}$ and $y_{t+1}$ denote the updated coordinates after one iteration; *v* is the step size or constant velocity that defines the distance traveled per time step; and $\theta _t$ corresponds to the current orientation angle of the agent, measured in radians relative to the horizontal axis. The trigonometric components $\cos (\theta _t)$ and $\sin (\theta _t)$ determine the displacement in the *x* and *y* directions, respectively, ensuring that the agent moves a fixed distance *v* per iteration in the direction defined by its current orientation.

Environmental perception is carried out in three directions ($\delta \in \{0,+\alpha ,-\alpha \}$) corresponding to the front, left, and right sensors, located at a distance *d* (Equation 2). Explorers use a larger *d*, while followers apply a reduced radius. In each direction, the values of mass (*M*), trail (*T*), and food (*F*) are obtained and weighted to generate an attractiveness score (Equation 3).

2 $$\begin{aligned} (x', y')= & \left( x + d \cdot \cos (\theta + \delta ),\ y + d \cdot \sin (\theta + \delta ) \right) \end{aligned}$$

3 $$\begin{aligned} \text {score}_i= & w_M \cdot M_i + w_T \cdot T_i + w_F \cdot F_i \end{aligned}$$

Where $(x', y')$ represents the coordinates of the sampling point used by the agent to sense the environment; (*x*, *y*) denotes the current position of the agent; *d* is the sensor offset distance from the agent’s center; $\theta$ is the current orientation of the agent; and $\delta$ is the angular offset that defines the direction of each sensor (left, forward, or right). This formulation allows each agent to probe the surrounding environment at three distinct angles relative to its orientation.

In Equation 3, $\text {score}_i$ represents the activation level or attraction score for sensor *i*, computed as a weighted sum of three environmental fields: $M_i$ corresponds to the local mass map, $T_i$ to the trail intensity, and $F_i$ to the food concentration detected at the sampling point. The parameters $w_M$, $w_T$, and $w_F$ are weighting coefficients that control the relative influence of each field on the agent’s decision-making process.

After moving, the agent deposits an amount of trail (*D*), dependent on food, the existing trail, and a minimum base (Equation 4). In addition, it contributes to the continuous mass map *M*(*x*, *y*), whose Gaussian smoothing (Equation 5) simulates the diffusion of cytoplasm in Physarum Polycephalum, highlighting regions of greater activity and growth fronts.

4 $$\begin{aligned} D= & \beta \cdot F + \gamma \cdot T + \varepsilon \end{aligned}$$

5 $$\begin{aligned} M_{\text {smooth}}= & \mathcal {G}_\sigma *M \end{aligned}$$

Where *D* represents the total amount of trail deposited by an agent at each iteration; *F* is the food field intensity at the agent’s current position; *T* is the local trail intensity previously accumulated in the environment; and $\varepsilon$ is a constant term representing the agent’s basal deposition rate. The parameters $\beta$ and $\gamma$ are weighting coefficients that control the influence of the food and existing trail fields, respectively, on the total deposition amount. This formulation reinforces trail accumulation in regions where both food and pheromone intensity are high, mimicking the adaptive reinforcement observed in the real *Physarum polycephalum* organism.

In Equation 5, $M_{\text {smooth}}$ denotes the smoothed version of the mass or trail map *M*, obtained by convolving it with a Gaussian kernel $\mathcal {G}_\sigma$ of standard deviation $\sigma$. The operator $*$ represents the convolution, which attenuates noise and preserves the main structural features of the network. This smoothing process approximates the continuous diffusion behavior of the slime mold, facilitating the visualization and analysis of the emergent patterns formed during the simulation.

The agent’s internal strength *s* decreases when no stimuli are found, according to Equation 6. If the hunger time exceeds the threshold $\tau _h$, it enters hibernation, representing local retraction. Reproduction, in turn, occurs selectively at the edges of the living mass, identified by morphological operations on $M_{\text {smooth}}$. If the agent has minimum strength, it can generate descendants with probability $p_{\text {rep}}$, transferring part of its energy and small variations in position and angle (Equation 7). This decentralized mechanism promotes adaptive expansion and mass redistribution of the system.

6 $$\begin{aligned} s_{t+1}= & \max (s_t - \delta , s_{\text {min}}) \end{aligned}$$

7 $$\begin{aligned} \mathbb {P}(\text {reproduction})= & p_{\text {rep}},\ \text {if } (x, y) \in \text {active frontier} \end{aligned}$$

Where $s_t$ represents the current activity or energy level of an agent at time step *t*; $s_{t+1}$ is the updated value after the reduction process; $\delta$ is the decay rate that determines how much the activity decreases per iteration; and $s_{\text {min}}$ is the minimum threshold value below which the agent cannot further reduce its state. This rule models the natural attenuation of agent activity in regions that are no longer reinforced, encouraging the contraction of inactive areas of the network over time.

In Equation 7, $\mathbb {P}(\text {reproduction})$ denotes the probability that a new agent is created at the current position, defined as a constant reproduction probability $p_{\text {rep}}$ applied only when the agent is located within the *active frontier*. This condition restricts reproduction to the expanding edges of the Physarum network, allowing growth to occur selectively in regions with ongoing exploratory activity, and preventing uncontrolled proliferation in saturated areas of the environment.

**Fig. 3:**
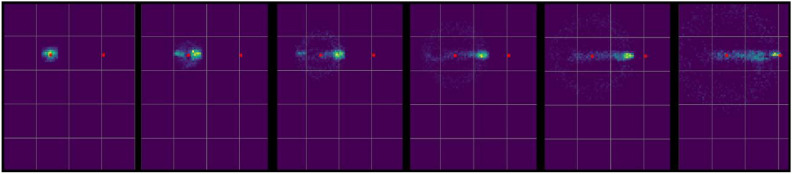
Visual evolution of the physarum network.

From the collective behavior of the agents, a network of stable trails emerges that connects the robots present in the environment. Figure 3 presents the visual evolution of this network over time, highlighting the system’s adaptation to the positions of the robots (red points on the map) and to the spatial distribution of stimuli.

### Emergent network formation and routing

Throughout the simulation, the movement of agents generates reinforced trails that, once stabilized, form an emergent high-conductivity network between the robots. To identify useful connections, the system selects points of higher trail intensity near each robot and applies a routing algorithm on the stabilized map. Thus, unlike traditional methods that depend on message exchange, the connections are implicitly inferred by the accumulated intensity of the explored paths, allowing each robot to locally determine its best physical links.

This process applies a routing algorithm on the stabilized map, identifying least-cost routes between the robots. The cost is defined from the trail map *T*(*x*, *y*), converted into a cost map *C*(*x*, *y*) by Equation 8, where reinforced trails correspond to lower traversal costs.

8 $$\begin{aligned} C(x, y) = \max (T) - T(x, y) + \varepsilon \end{aligned}$$

Where *C*(*x*, *y*) represents the local cost of traversal at position (*x*, *y*), computed as the inverse of the trail intensity to promote path selection along the most reinforced regions of the map. *T*(*x*, *y*) denotes the trail intensity at the corresponding position, and $\max (T)$ is the maximum trail value in the entire map, used for normalization. The constant $\varepsilon$ is a small positive offset that prevents division by zero or null cost values. In this formulation, higher trail intensities result in lower traversal costs, guiding the emergent network to favor routes with stronger reinforcement and more stable connectivity.

The function is applied between two points considering weights given by *C*(*x*, *y*), resulting in a path of minimum accumulated cost. The average trail intensity along this path, calculated by Equation 9, defines the quality of the connection, with the one of highest value being selected as dominant.

9 $$\begin{aligned} \bar{T}_{\text {path}} = \frac{1}{N} \sum _{(x_i, y_i) \in \text {path}} T(x_i, y_i) \end{aligned}$$

Where $\bar{T}_{\text {path}}$ represents the average trail intensity along a specific path connecting two points of interest; $T(x_i, y_i)$ is the trail intensity at each discrete coordinate $(x_i, y_i)$ belonging to the selected path; and *N* is the total number of sampled points along that path. This metric provides a quantitative measure of the overall reinforcement accumulated along a route, allowing comparison between different candidate paths in terms of their stability and strength within the emergent Physarum network.

In this way, the communication network is formed autonomously, locally, and in a biologically plausible manner, continuously adapting to environmental conditions and task dynamics. Scientific references support the biological plausibility of the decisions implemented^26,27^. The result is an emergent network formation system that can be applied to connectivity, routing, and coordination problems in decentralized multi-robot systems.

Beyond the biological plausibility, the proposed mechanism offers a significant advantage in terms of communication complexity. In traditional flooding-based MANET protocols, route discovery often involves broadcasting messages to all neighbors, leading to a message complexity of $O(N^2)$ in the worst case, where *N* is the number of nodes. In contrast, the Physarum-inspired layer operates locally to filter the network topology based on the cost map *C*(*x*, *y*). By restricting active data exchange (task sharing) only to the reinforced trails, which represent a sparse subset of the available physical links, the algorithm limits the effective node degree to a smaller average *k* (where $k \ll N$). Consequently, the communication overhead for task synchronization scales linearly, approximately $O(k \cdot N)$, ensuring scalability and minimizing bandwidth usage even as the number of robots increases. This implies that the computational effort of the Physarum algorithm directly translates into reduced network traffic, preventing the ‘broadcast storm’ problem typical in dense robotic networks.

### Simulation environment

**Fig. 4:**
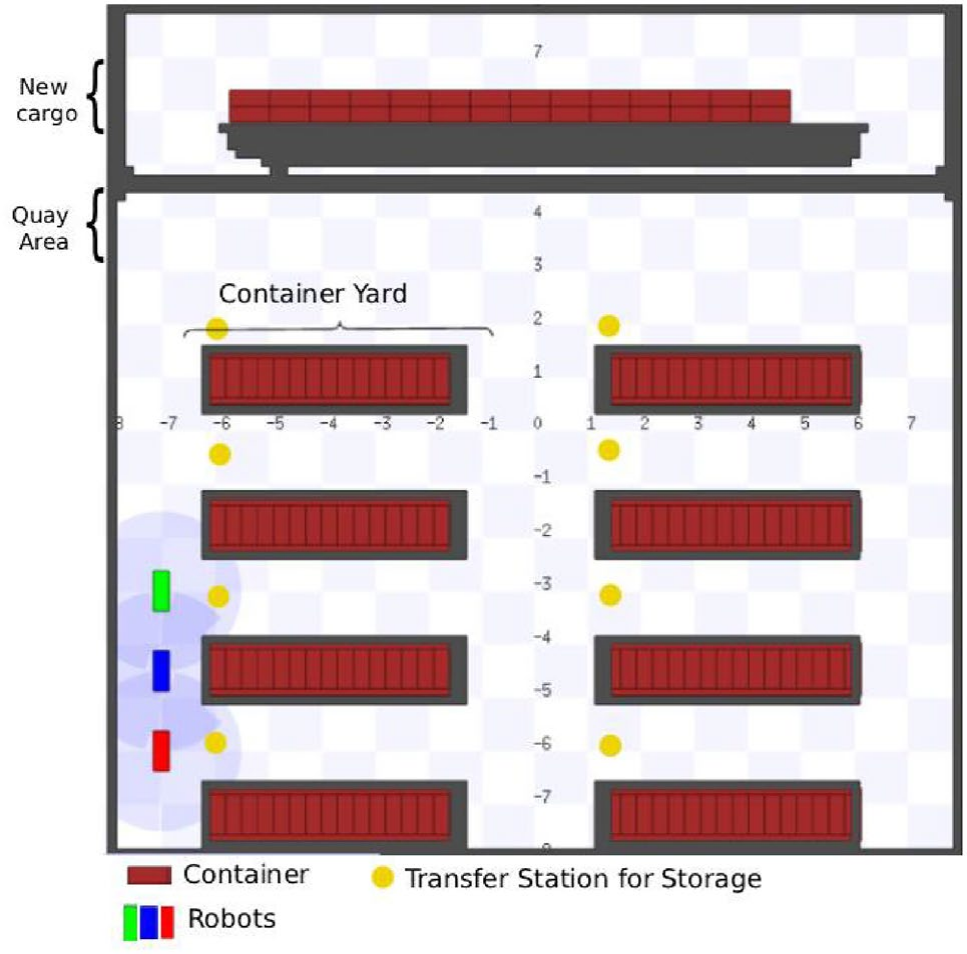
Structure of the simulated port scenario.

To evaluate the functioning of the proposed algorithm, a simulation environment was developed that represents a logistics space of a port shared by multiple autonomous mobile robots, subject to operational constraints and signal interference. This environment was implemented using the Stage platform, integrated with ROS. Stage was chosen primarily for its computational efficiency and deterministic behavior, which are essential for studying decentralized coordination and network formation under controlled conditions. Unlike other simulators, Stage provides a lightweight 2D environment that allows precise control over spatial configuration and experiment repeatability, without the complexity of a full physical simulation. In Fig. 4, the proposed scenario can be visualized.

**Fig. 5:**
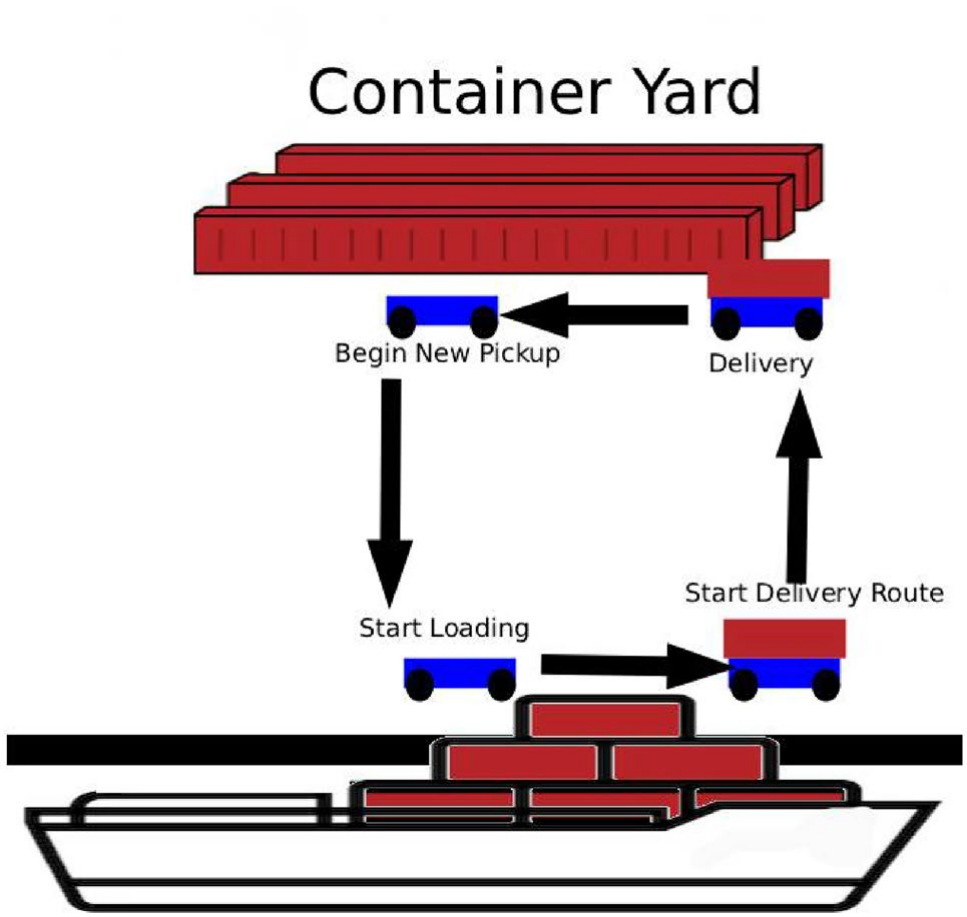
Container unloading process simulated in this work.

The mobile robots, represented in green, blue, and red, move through the environment performing logistics tasks and using routing strategies inspired by Physarum Polycephalum, adapting to the spatial distribution of containers and to changes in available routes. In this context, the containers play a dual role: they act as loads to be handled and also as physical obstacles that interfere with robot connectivity, simulating signal blocking and attenuation, a common phenomenon in real port operations. Figure 5 presents the container unloading process carried out in the simulation of a port terminal.

Specifically, information exchange between two robots is only allowed when the algorithm based on Physarum Polycephalum identifies that there is an active connection between them, evidenced by the formation of a reinforced trail in the environment. When such a connection is detected, a ROS topic is temporarily used to enable information sharing between the connected robots. Outside these detected connectivity moments, the robots operate in isolation, without direct message exchange.

## Experiments and evaluation

The experimental evaluation aimed to systematically verify the performance and feasibility of the connectivity algorithm inspired by Physarum Polycephalum applied to decentralized coordination of mobile robots. Four complementary experiments were conducted, designed to analyze different aspects of the system in a simulated logistics environment, integrating autonomous navigation, selective information exchange, and task distribution.

### Experiment 1 – evaluation of the Physarum polycephalum network: connectivity

The first validation carried out was the network connectivity assessment, that is, the ability of the robots to establish connections in order to achieve global connectivity among all robots. The hypothesis tested considers that robots, using only local perception, would be able to detect the presence of other robots and progressively form connections until all are interconnected through a decentralized mesh. For this evaluation, five distinct scenarios were defined, with different initial robot positions on the map. In each scenario, the robots were positioned in regions where mutual detection was possible, respecting the signal range limits of each robot. Each scenario was repeated five times, totaling 25 independent runs.

Table 1 presents a summary of the results obtained, with the pairs of connected robots and the respective average connection times per scenario. It is observed that, in all tests performed, the three robots were able to establish a connection within up to two seconds after the start of the simulation. The overall average connection times remained close to 1 second, with expected variations related to small differences at the beginning of interaction and the local perception of the agents.

**Table 1:** Summary of connectivity results per scenario (average of 5 runs).

Scenario	Robot | Position	Connected Robot	Average Time (s)
1	robot_0 (2.0, −0.4)	robot_1	0.86
	robot_1 (−2.2, −0.4)	robot_2	1.28
	robot_2 (0.0, 2.8)	robot_1	1.29
2	robot_0 (0.0, −3.1)	robot_1	1.29
	robot_1 (−2.2, −0.4)	robot_2	1.10
	robot_2 (0.0, 2.8)	robot_1	0.91
3	robot_0 (0.0, −3.1)	robot_1	0.86
	robot_1 (2.4, −3.2)	robot_2	1.09
	robot_2 (1.6, −0.4)	robot_0	1.23
4	robot_0 (0.0, −3.1)	robot_1	1.46
	robot_1 (2.4, −3.2)	robot_0	0.83
	robot_2 (0.0, 2.8)	robot_0	1.07
5	robot_0 (4.5, −5.8)	robot_2	0.98
	robot_1 (2.4, −3.2)	robot_2	0.91
	robot_2 (6.5, −3.1)	robot_0	0.72

In decentralized mobile robot logistics systems, the decision and connection time between units must be significantly lower than the physical travel time or the execution time of transport tasks. Since the robots in this simulation take longer to complete a pickup or delivery task, a connection time of 1 to 2 seconds is sufficient for the communication mesh to stabilize and for task exchange to occur before any critical movement decision.

Topological consistency was also observed: the connection pairs repeated across the runs of the same scenario, indicating stability in the organization of the network.

The results obtained confirm that the proposed algorithm is capable of establishing connections efficiently and in a decentralized manner, even in different spatial configurations.

This experiment demonstrated, with quantitative evidence (average connection times around 1 second, variations between scenarios, and repeatability in 25 runs) and qualitative evidence (topological consistency of established connections, absence of connectivity fault, and progressive mesh formation in all runs), that the proposed algorithm is capable of establishing networks with full connectivity, acceptable response time, and stable topology.

### Experiment 2 – Evaluation of the Physarum polycephalum network: robustness

**Fig. 6:**
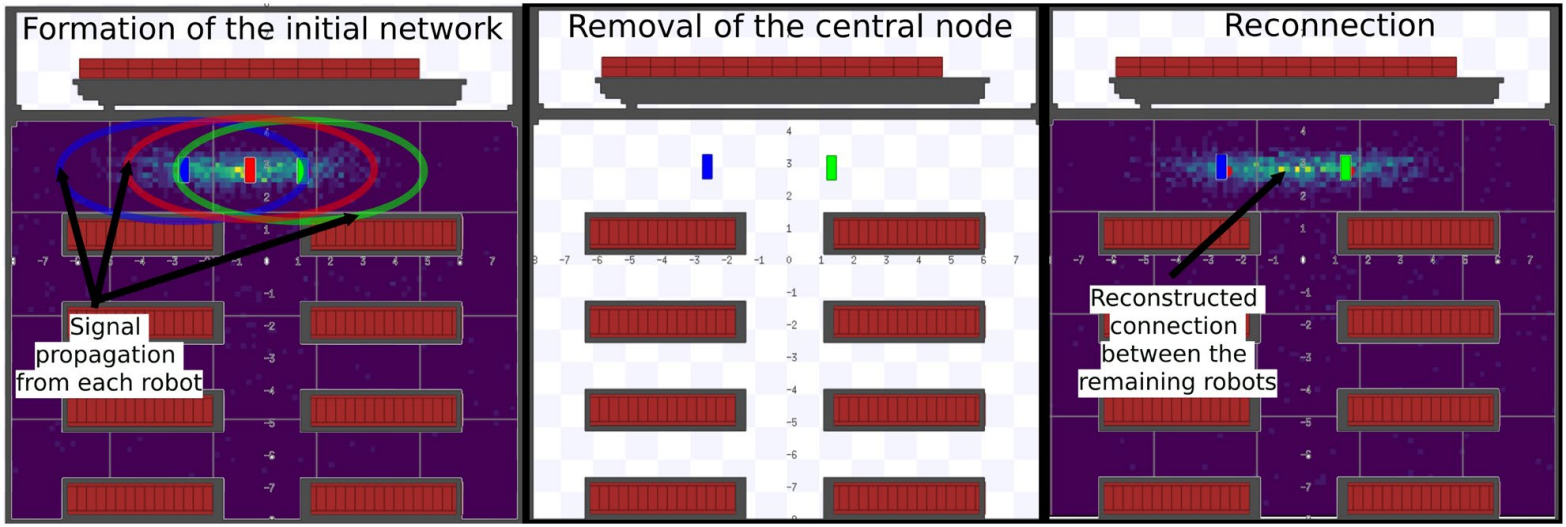
Mesh network reconnection scenario.

This stage of the experimental evaluation focused on analyzing the robustness of the connectivity algorithm in the face of robots fault in the network. In mobile robotics systems, the ability of the system to reconfigure and maintain communication is crucial to ensure the continuity of logistical operations. The objective of this experiment was to verify whether the removal of a central robot, which served as a connection link for other robots, would result in a new direct connection between the remaining robots without the need for external intervention. A representation of the experiment can be observed in Fig. 6

For this test, a specific scenario was designed where one robot acted as a common connection point between two others.

The experiment was conducted in three controlled stages:

1. Initial Network Formation: The three robots were positioned, ensuring that the signal range of each was sufficient so that all could connect. The simulation was started, and the connectivity algorithm inspired by Physarum Polycephalum was activated so that the mesh network could stabilize. Initial connection data and formation time were recorded, in the same way as in Experiment 1.
2. Central Robot Removal: After the network was fully formed and stable, the central robot (or “pivot” robot) was removed from the simulation. This removal simulated an abrupt robot failure, such as battery loss or a mechanical problem.
3. Reconnection Analysis: The simulation continued so that the two remaining robots could react to the loss of the central robot. The analysis focused on two aspects: the ability of the remaining robots to detect the absence of their previous partner and, more importantly, the formation of a new direct connection between them. The time required for this reconnection to occur was recorded from the moment of the central robot’s removal.

For greater stability and consistency of results, this scenario was repeated 9 times, with the same robot configuration repeated every 3 runs but in different positions on the map. The results are presented in Table 2. The results compiled in Table 2 demonstrate the consistency of the algorithm in restoring connectivity. In all 9 runs, reconnection was successful, with an average time of 0.001 seconds, a value that amounts to an instantaneous reconnection. This result confirms the robustness of the model in robots fault scenarios.

**Table 2:** Robustness experiment results.

Run	Initial Connection	Central Robot Removed	Reconnection Time (s)	New Connection
1	r1-r0-r2	robot_0	0.001	r1 $\leftrightarrow$ r2
2	r0-r1-r2	robot_1	0.001	r0 $\leftrightarrow$ r2
3	r0-r2-r1	robot_2	0.001	r0 $\leftrightarrow$ r1
4	r1-r0-r2	robot_0	0.001	r1 $\leftrightarrow$ r2
5	r0-r1-r2	robot_1	0.001	r0 $\leftrightarrow$ r2
6	r0-r2-r1	robot_2	0.001	r0 $\leftrightarrow$ r1
7	r1-r0-r2	robot_0	0.001	r1 $\leftrightarrow$ r2
8	r0-r1-r2	robot_1	0.001	r0 $\leftrightarrow$ r2
9	r0-r2-r1	robot_2	0.001	r0 $\leftrightarrow$ r1
**Average**			**0.001**	

The extremely low reconnection time can be explained by the nature of the linear network topology used in these tests. Since the remaining robots were already within each other’s signal range, the removal of the central robot did not require a search and discovery process for a new partner. Instead, the fault of the central robot was immediately perceived, and the direct connection between the robots was established instantly, as they were already detecting each other in their field of view. In essence, the algorithm did not need to “search” for a new connection, it simply activated a potential connection that already existed. This consistency in results suggests that the algorithm is reliable in reconfiguring the communication mesh.

### Experiment 3 – Evaluation of the Physarum polycephalum network: adaptability

**Fig. 7:**
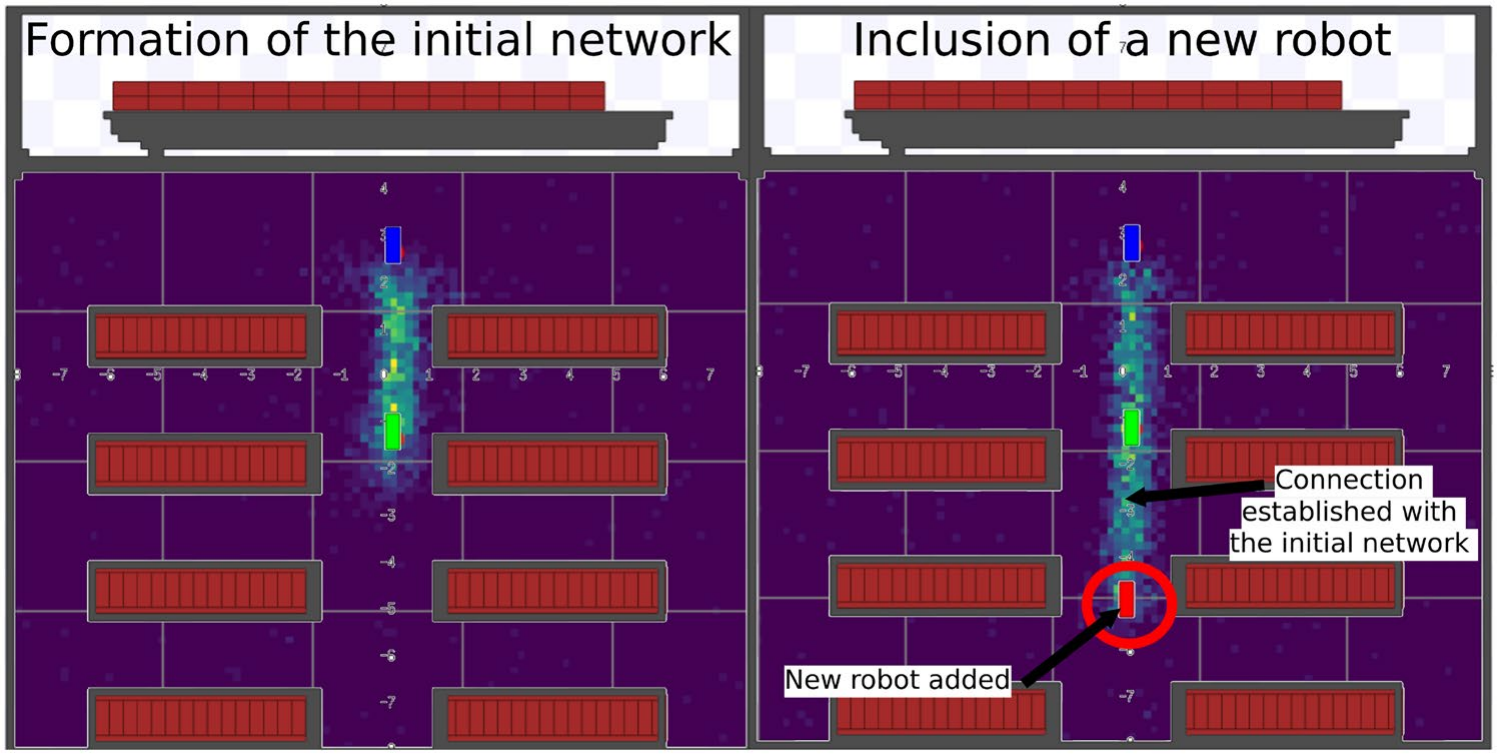
Scenario of a robot connecting to an existing mesh network.

This experimental stage focused on the adaptability of the algorithm to include new robot in an already established communication mesh. In dynamic logistics environments, the entry of new robots into the operating area is a common event, and their ability to autonomously integrate into the network is fundamental for the scalability and efficiency of the system. A representation of the experiment can be observed in Figure 7

The experiment was divided into two stages:

1. Initial Network Formation: Two robots (r1 and r2) were positioned to establish a direct connection and form the base of the network. The simulation was started and the connection was monitored until it was fully stabilized.
2. Inclusion of a New Robot: After the initial network was stable, a third robot (r0) was introduced into the signal field of one of the already connected robots. The analysis focused on the time the new robot took to detect the network and establish its first connection, integrating into the mesh.

This scenario was repeated 5 times, with the third robot being introduced in different positions, to verify whether connection performance varied with distance or initial position. Table 3 presents the preliminary results obtained for this experiment, highlighting the agility of the algorithm in incorporating a new robot.

**Table 3:** Adaptability experiment results.

Run	Initial Network	Robot Added	Connection Time (s)
1	r1 $\leftrightarrow$ r2	r0	0.10
2	r1 $\leftrightarrow$ r2	r0	0.08
3	r1 $\leftrightarrow$ r2	r0	0.09
4	r0 $\leftrightarrow$ r1	r2	0.10
5	r0 $\leftrightarrow$ r1	r2	0.09
6	r0 $\leftrightarrow$ r1	r2	0.09
7	r0 $\leftrightarrow$ r2	r1	0.10
8	r0 $\leftrightarrow$ r2	r1	0.09
9	r0 $\leftrightarrow$ r2	r1	0.09
**Average**			**0.092**

Analyzing the data from Table 3, the results consistently demonstrate the agility and efficiency of the connectivity algorithm in integrating new robots into the mesh network, with an average connection time of 0.092 seconds. This result is significant and highlights a crucial point of the algorithm inspired by Physarum Polycephalum: its capacity for expansion and scalability. The rapid incorporation of a new robot into the network, without disturbing the existing connection, is evidence that the model can adapt to dynamic environments where robots enter and leave the operating area.

### Experiment 4 - task distribution

This experiment had as its main objective to verify the practical impact of the bio-inspired connectivity algorithm on the feasibility of a decentralized coordination system. For this purpose, a simulated scenario was used that integrates autonomous robot navigation with the distribution of logistical tasks. The focus was to quantify the performance improvement obtained when robots are able to efficiently exchange information through the Physarum Polycephalum network.

For this experiment, two test scenarios were created, identical in all parameters except for the presence of the connectivity algorithm and information exchange.

The task distribution scenario without communication served as the control group. In this scenario, the robots operated completely in isolation, without any type of communication between them. Task distribution occurred in a simplified manner: tasks were assigned to each robot in a fixed and sequential way before the start of the simulation. Upon completing a task, each robot processed the next one from its own local queue. The absence of information exchange prevented the robots from having knowledge about each other’s workload or about the location of tasks that could be redistributed. Consequently, there was no possibility of load balancing. The system’s performance was evaluated based on the total time required to complete all tasks and on the analysis of each robot’s workload, which was expected to be uneven.

To establish a clear comparison baseline, the experimental scenario was executed five times. In each repetition, a total of 15 tasks was distributed intentionally unevenly among the three robots, simulating a scenario of non-optimized task allocation. This strategy aimed to create a workload imbalance, resulting in idle moments for some robots while others were still processing their tasks. The collected data detail the completion time of each robot and the total execution time of the simulation, which corresponds to the time required for the most overloaded robot to complete its last task. Table 4 summarizes these results. The average total execution time was estimated from the highest time recorded by a robot in each run. Thus, it is considered that the total time is only reached when all tasks have been completed.

**Table 4:** Scenario 1 results: task distribution without communication.

Run	Number of Tasks			Robot Completion Time (s)		
	r0	r1	r2	r0	r1	r2
1	7	5	3	685	436	258
2	4	5	6	572	600	674
3	5	5	5	477	381	409
4	2	3	10	213	324	690
5	6	6	3	579	511	252
**Average Total Execution Time (s)**				621		

With this, the same tests were applied, but now using information exchange between the robots through the connection established by the algorithm inspired by Physarum Polycephalum during operation. The mesh network formed by the Physarum Polycephalum algorithm was used so that the robots could share, in a decentralized manner, data about their current workload and task locations. Upon connecting, the robots exchanged messages that enabled task sharing. Table 5 presents the results obtained from task execution in a scenario with sharing.

From the perspective of a single robot, each connection event triggers a short data-exchange phase. The robots broadcast a summary of their local task lists, including task identifiers, status (pending, executing, or completed), and timestamps of the last update. Upon reception, each robot compares the received entries with its own queue: duplicated tasks are removed, outdated entries are updated, and new tasks are appended if not already assigned. This lightweight reconciliation uses the latest timestamp as the tie-breaker, ensuring that the most recent information prevails. When disconnection occurs, each robot continues executing its current queue independently, keeping local logs of all task state transitions. When future reconnections take place (even with different neighbors), this history allows robots to re-synchronize and avoid redundant execution. Although simplified, this local timestamp-based mechanism prevents inconsistencies and redundant task assignments under temporary network fragmentation.

**Table 5:** Scenario 2 results: task distribution with communication.

Run	Number of Tasks			Robot Completion Time (s)		
	r0	r1	r2	r0	r1	r2
1	7	5	3	531	434	484
2	4	5	6	503	531	524
3	5	5	5	459	438	474
4	2	3	10	513	484	501
5	6	6	3	381	397	402
**Average Total Execution Time (s)**				510		

The analysis of the results from both test scenarios allows for a direct comparison of the impact of the connectivity algorithm on efficiency and load balancing. While Scenario 1 (without connectivity) established the baseline for a simplified system, Scenario 2 (with the Physarum Polycephalum-inspired algorithm) demonstrated how decentralized information exchange can optimize operation. In Scenario 1, the average total execution time was 621 seconds. This result was expected, as the system depended on the most overloaded robot to complete its tasks, while the other robots could remain idle. In Scenario 2, the average total execution time was reduced to 510 seconds. This reduction of 111 seconds represents an improvement of approximately 17.87% in the overall efficiency of the system. The decrease in total execution time validates the hypothesis that the connectivity established by the Physarum Polycephalum-inspired algorithm is sufficient to enable efficient information exchange, resulting in superior performance. In addition to the improvement in total time, the analysis of the individual completion times of each robot reveals the success of the algorithm in load balancing.

## Conclusion

The present work had as its main objective to demonstrate the feasibility and performance of a decentralized coordination solution for multi-robot systems, inspired by the self-organization of Physarum polycephalum. The proposal was based on an architecture that allows robots to form and maintain a dynamic communication network, adapting to topological changes and system fault. From the experiments conducted in a simulated environment, it was possible to address the central research questions, proving the effectiveness and potential of the approach.

The hypothesis that robots, operating with purely local perception, would be capable of forming a functional mesh network and achieving global connectivity was validated. The experimental results showed that the proposed algorithm is responsive, establishing connections in an average time of less than two seconds. This agility is crucial in dynamic environments, where the rapid formation of the communication network is a prerequisite for cooperation.

The robustness of the network in the face of fault, another fundamental question, was also confirmed. The experiments demonstrated the ability of the network to reconfigure almost instantly after the removal of a central robot. This behavior highlights the resilience of the algorithm, ensuring the continuity of operation even in the face of sudden agent losses, an indispensable attribute for autonomous systems in real contexts.

The adaptability of the algorithm was evidenced through experiments, which showed the network’s ability to absorb new robots quickly and efficiently, with an average connection time of 0.092 seconds. This finding validates the scalability potential of the solution, suggesting that the model can expand to include new robots without compromising the stability or performance of the existing communication mesh.

Finally, the hypothesis that the established connectivity would be sufficient to enable load balancing and improve system efficiency was proven through the proposed experiments. The comparison between scenarios with and without communication showed a substantial improvement in total execution time, from 621 to 510 seconds, representing a 17.87% efficiency gain. The detailed analysis revealed that this improvement is a direct result of dynamic load balancing, which transformed the unequal performance of robots into a more uniform cooperative effort. The apparent increase in the individual times of some robots actually reflects the transfer of tasks from overloaded agents to idle ones, optimizing the overall system performance.

In conclusion, the results obtained in this work solidify the proposal of a bio-inspired solution as a viable and robust alternative for decentralized coordination in multi-robot systems. The algorithm proved capable of generating efficient, resilient, and adaptable connectivity networks, which serve as the basis for information exchange and cooperation among agents.

## Future work

Future research directions include expanding the scalability analysis to scenarios with a larger number of robots and validating the proposed approach in a physical environment to demonstrate its applicability under real operating conditions.

Future studies may also explore the integration of the Physarum-inspired connectivity layer with established communication frameworks such as MANET. While not implemented in the current study, this conceptual alignment opens the possibility of using the emergent link-weighting process to enhance routing efficiency and quality-of-service adaptation in robotic networks.

In addition, future research should include a systematic analysis of communication complexity and scalability. While this work focused on validating connectivity and decentralized coordination through simulation, the behavior of the proposed mechanism under real-world communication constraints, such as signal interference, latency variability, and hardware limitations, remains to be investigated. Evaluating how message exchange scales with the number of robots, link density, and environmental factors will provide valuable insights into potential latency, bandwidth usage, and performance bottlenecks in larger and more realistic robotic networks.

## Data Availability

The data are available in the manuscript or on GitHub: https://github.com/mdieisson/Physarum_ws
